# Effects of problem-focused versus emotion-focused behavioral interventions on peptic ulcer recurrence: a 4-year retrospective cohort study

**DOI:** 10.3389/fmed.2026.1820736

**Published:** 2026-06-16

**Authors:** Haiyan Peng, ShaoXue Li

**Affiliations:** The Third people’s Hospital of Hefei, Hefei, China

**Keywords:** behavioral intervention, emotion-focused coping, peptic ulcer recurrence, problem-focused coping, retrospective cohort study

## Abstract

**Background:**

Peptic ulcer disease (PUD) exhibits high recurrence rates, and behavioral interventions targeting coping strategies may influence long-term outcomes. However, the differential associations of problem-focused versus emotion-focused structured interventions with ulcer recurrence remain unclear.

**Objective:**

To evaluate the association between problem-focused versus emotion-focused behavioral interventions delivered during hospitalization and subsequent 4-year ulcer recurrence in patients with PUD.

**Methods:**

This single-center retrospective cohort study included 167 patients hospitalized with PUD between January 2021 and March 2025. Based on documented structured interventions received during the index hospitalization, patients were classified into a problem-focused intervention group (*n* = 84; 6 monthly 90-min group sessions emphasizing problem identification, solution generation, goal-setting, and action planning) or an emotion-focused intervention group (*n* = 83; 6 monthly 90-min group sessions focusing on emotional awareness, relaxation training, mindfulness, and cognitive restructuring). Clinical outcomes and endoscopic findings were ascertained retrospectively from the electronic medical record over a 4-year period (January 2021 to March 2025). Baseline demographic and clinical characteristics were comparable between groups. Outcome assessors (endoscopists) were blinded to group assignment; patient blinding was not feasible given the behavioral intervention context.

**Results:**

Over the 4-year observation period, cumulative ulcer recurrence was significantly lower in the problem-focused group (19.05% vs. 37.35%; χ^2^ = 7.243, *P* = 0.007). After multivariable adjustment for prespecified confounders (baseline ulcer diameter, NSAID use history, smoking status, *H. pylori* positivity, age, and gender), problem-focused intervention remained independently associated with reduced odds of recurrence (adjusted OR = 0.41, 95% CI: 0.22–0.76, *P* = 0.004). The problem-focused group also demonstrated higher intervention adherence (92.86% vs. 81.93%, *P* = 0.038), greater patient satisfaction (22.41 ± 2.30 vs. 19.75 ± 2.98, *P* < 0.001), and shorter median healing time (6.0 vs. 7.0 weeks, *P* = 0.022). No significant differences were observed in *H. pylori* reinfection rates or adverse events.

**Conclusion:**

In this retrospective cohort, receipt of a structured problem-focused behavioral intervention during hospitalization was associated with lower ulcer recurrence and improved clinical outcomes over the 4-year observation period compared to an emotion-focused intervention. However, given the observational design, single-center setting, lack of participant blinding, and potential residual confounding, these findings should be interpreted as hypothesis-generating. Prospective randomized controlled trials are warranted to establish causality and evaluate generalizability before integration into routine clinical practice.

## 1 Introduction

Peptic ulcer disease (PUD) represents a prevalent chronic and recurrent condition in clinical practice. Its pathogenesis is closely associated with aberrant gastric acid secretion, *Helicobacter pylori* (*H. pylori*) infection, and compromised mucosal defense mechanisms (1). Ulcer recurrence constitutes a major therapeutic challenge, as persistent episodes not only exacerbate patient discomfort but also impose a substantial healthcare burden (2). In recent years, the role of psychosocial and behavioral factors in the onset and progression of PUD has garnered increasing attention. Notably, the specific type of coping strategy employed by patients may influence ulcer healing and recurrence rates through activation of the hypothalamic-pituitary-adrenal (HPA) axis and subsequent cortisol release, which can modulate gastric acid secretion and compromise mucosal defense mechanisms (3). It is important to distinguish between an individual’s inherent coping style—a relatively stable dispositional tendency—and the receipt of structured behavioral interventions designed to cultivate specific coping skills. Coping style reflects how a person typically responds to stressors, whereas coping-focused interventions represent an active attempt to modify coping behaviors through psychoeducation and skills training. This study investigates the latter: we examine the association between receiving a structured behavioral intervention grounded in problem-focused principles (emphasizing proactive problem identification, solution generation, and action planning) versus an intervention grounded in emotion-focused principles (emphasizing emotional awareness, relaxation, and cognitive reappraisal) and long-term clinical outcomes. Problem-focused interventions aim to equip patients with active self-management skills that may reduce chronic stress exposure and its physiological consequences, including HPA axis dysregulation and impaired mucosal defense. Emotion-focused interventions primarily target the regulation of negative affective states without directly addressing the external stressors that may perpetuate disease processes (4). Long-term follow-up is theoretically essential because stress-induced HPA axis activation and its downstream effects on acid secretion and mucosal integrity are cumulative processes; sustained exposure to elevated cortisol levels over months or years may progressively impair mucosal healing capacity and increase vulnerability to ulcer recurrence. PFC may be more advantageous for improving clinical outcomes in patients with functional gastrointestinal disorders; however, its role in organic diseases such as PUD remains inadequately characterized (5).

Although several studies have examined the impact of psychological and behavioral interventions on PUD prognosis, findings have been mixed. Some randomized trials suggest that stress management or coping skills training may improve symptom control and quality of life, while others have found no significant benefit over usual care (6). A systematic comparison of different coping-focused intervention approaches is lacking in the current literature, particularly regarding long-term follow-up data incorporating objective clinical outcomes (7). Existing research frequently relies on subjective psychological measures as primary endpoints and seldom includes endoscopic confirmation of ulcer recurrence, standardized gastrointestinal symptom scores, and validated quality-of-life assessments as integrated outcomes (8–10). Moreover, many studies are limited by small sample sizes, heterogeneous intervention protocols, and insufficient control for potential confounding variables (11–13). Consequently, whether one type of structured coping-focused intervention is associated with more favorable long-term PUD outcomes than another remains an open question Critically, the mechanistic pathways through which coping strategies might influence ulcer recurrence remain poorly delineated. Key questions persist regarding whether these effects are mediated through enhanced treatment adherence, attenuation of HPA axis-mediated acid hypersecretion, or potentiation of mucosal repair processes (14, 15).

While coping style is often considered a patient trait, it can be modified through structured training. This retrospective study aimed to evaluate the association between two such structured behavioral interventions—one based on problem-focused coping (PFC) principles and the other on emotion-focused coping (EFC) principles—and long-term ulcer recurrence. We hypothesized that the problem-focused intervention, by equipping patients with active self-management skills, would be associated with a lower recurrence rate compared to the emotion-focused intervention over a 4-year observation period. Adhering to the Strengthening the Reporting of Observational Studies in Epidemiology (STROBE) guidelines for cohort studies, this retrospective investigation utilized data collected from January 2021 to March 2025, incorporating a multidimensional assessment strategy including endoscopic evaluation, validated symptom and quality of life scales, and adherence measures. Multivariable regression was used to control for potential confounders. Multivariable regression was used to control for potential confounders.

## 2 Materials and methods

### 2.1 General information

**FIGURE 1:**
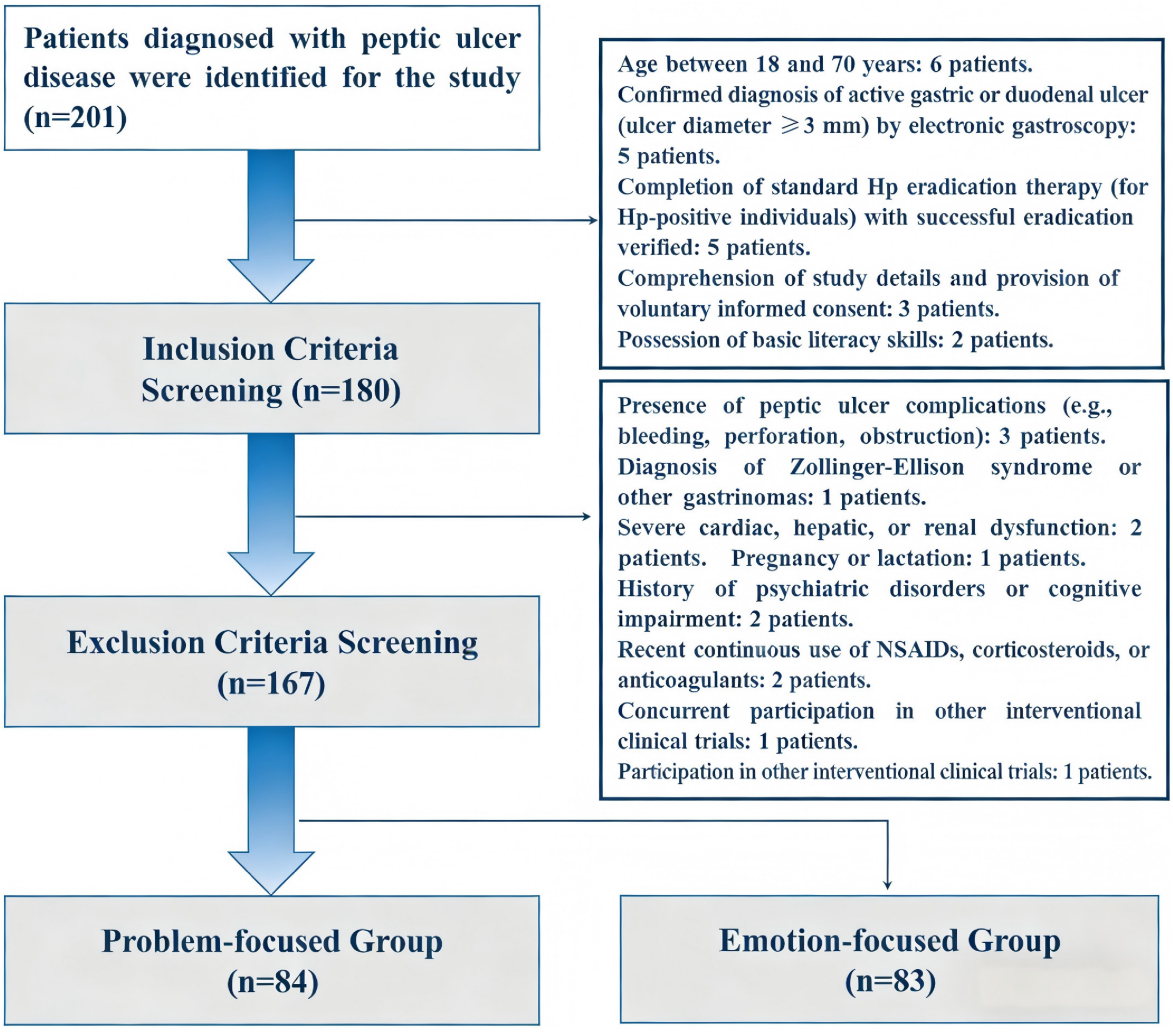
Research roadmap.

This was a single-center retrospective cohort study designed to evaluate the association between two distinct in-hospital behavioral interventions and subsequent ulcer recurrence over a 4-year outcome ascertainment window (January 2021 to March 2025). The study protocol received approval from the Institutional Review Board of our hospital (Ethics Code 2026LLWL016). The requirement for written informed consent was waived due to the retrospective, anonymized nature of the data analysis, in accordance with institutional and national regulations for secondary use of clinical data. The study is reported in accordance with the Strengthening the Reporting of Observational Studies in Epidemiology (STROBE) guidelines for cohort studies. We reviewed the medical records of patients hospitalized for peptic ulcer disease at our institution between January 2021 and March 2025. A total of 167 patients met the inclusion criteria and had complete medical records documenting the structured coping intervention they received during hospitalization. Based on the intervention documented in their records, patients were categorized into either a problem-focused intervention group (*n* = 84) or an emotion-focused intervention group (*n* = 83). The cohort comprised 120 males (71.9%) and 47 females (28.1%), with a mean age of 47.3 ± 10.8 years. No statistically significant differences (*P* > 0.05) were observed between the two groups regarding baseline characteristics, including age, gender distribution, ulcer type, and initial *Helicobacter pylori* (Hp) infection rate, confirming their comparability at baseline. The study design and reporting follow the Strengthening the Reporting of Observational Studies in Epidemiology (STROBE) guidelines for cohort studies. The research route is shown in Figure 1.

### 2.2 Inclusion and exclusion criteria

Inclusion criteria: (1) Age between 18 and 70 years; (2) Confirmed diagnosis of active gastric or duodenal ulcer (ulcer diameter ≥ 3 mm) by electronic gastroscopy; (3) For patients with pre-eradication *H. pylori* positivity (documented by 13C-urea breath test, rapid urease test, or histology at the time of index ulcer diagnosis), completion of standard 14-day bismuth-containing quadruple eradication therapy prior to enrollment, with successful eradication verified by a negative 13C-urea breath test (DOB < 4.0) performed at least 4 weeks after completion of eradication therapy and at least 2 weeks after discontinuation of proton pump inhibitors/potassium-competitive acid blockers and at least 4 weeks after discontinuation of antibiotics and bismuth compounds, in accordance with the Sixth Chinese National Consensus Report on *H. pylori* Infection Management; (4) Comprehension of the study details and provision of voluntary informed consent; (5) Possession of basic literacy skills sufficient to complete questionnaire-based assessments. Consequently, all patients included in the final cohort were *H. pylori*-negative at enrollment (confirmed eradication).

Exclusion criteria: (1) Presence of peptic ulcer complications (e.g., bleeding, perforation, obstruction); (2) Diagnosis of Zollinger-Ellison syndrome or other gastrinomas; (3) Severe cardiac, hepatic, or renal dysfunction (Child-Pugh score ≥ B grade or eGFR < 30 ml/min/1.73 m^2^); (4) Pregnancy or lactation; (5) History of psychiatric disorders or cognitive impairment; (6) Recent (within 1 month) continuous use of Non-Steroidal Anti-Inflammatory Drugs (NSAIDs), corticosteroids, or anticoagulant medications; (7) Concurrent participation in other interventional clinical trials.

### 2.3 Equipment and materials

(1) Electronic Gastroscopy System: High-definition Olympus GIF-H290 electronic gastroscopes coupled with MISS image processing systems were utilized for baseline ulcer diagnosis and recurrence assessment during follow-up. All endoscopic procedures were performed by two senior attending physicians, with diagnostic consistency confirmed by a Kappa value of 0.87 (indicating excellent agreement).

(2) *Helicobacter pylori* infection was detected using the HY-IREXB type 13C urea analyzer (Guangzhou Huayou Mingkang Optoelectronic Technology Co., Ltd.) along with its accompanying reagent kit. Participants were required to fast for a minimum of 4 h before the test. After oral administration of 13C-urea diagnostic reagent, respiratory samples were collected at 0 min (baseline) and 30 min post-ingestion. The device automatically computed and reported Delta Over Baseline (DOB) values, with a DOB ≥ 4.0 considered positive for *H. pylori* infection. In accordance with the Sixth Chinese National Consensus Report on *H. pylori* Infection Management, proton pump inhibitors and potassium-competitive acid blockers were withheld for at least 2 weeks, and antibiotics and bismuth compounds were withheld for at least 4 weeks, prior to all 13C-urea breath testing to minimize false-negative results. *H. pylori* testing was performed at the following time points: (i) initial diagnosis (at index endoscopy); (ii) confirmation of eradication (≥ 4 weeks post-eradication therapy, median 6 weeks, prior to enrollment); (iii) annual follow-up assessments during the 4-year observation period.

(3) Biochemical analysis system: Serum parameters (including liver and kidney functions and lipid profile) were detected by Siemens XP biochemical analyzer. The whole blood cell technology was accomplished using Mindray 5,390 and 6,900 blood analyzers.

(4) Electronic data capture system: Questionnaire data were collected via iPad 9th Generation tablets pre-installed with Research Electronic Data Capture (RedCap) software, enabling real-time upload to secure servers to minimize entry errors.

Of note, as part of our institution’s standardized admission protocol for patients with newly diagnosed or complicated peptic ulcer disease, comprehensive laboratory evaluation including serum gastrin-17 and pepsinogen I/II ratio is routinely performed to rule out Zollinger-Ellison syndrome and assess gastric mucosal functional status. This standardized approach ensured the consistent availability of these parameters in the medical records of all included patients.

### 2.4 Research methods

(1) Implementation of the intervention protocol

(1) Description of the structured interventions. During the index hospitalization, patients received one of two standardized, multicomponent psychoeducational programs delivered in a group-based format. Both programs were designed to provide equivalent contact time and structure: 6 monthly sessions of 90 min each, supplemented by illustrated take-home manuals. Sessions were facilitated by uniformly trained gastroenterology specialist nurses and clinical psychologists. It is important to note that both interventions were active, manualized programs; the study does not compare an intervention to a no-treatment control, but rather examines the differential associations of two theoretically distinct active interventions with long-term outcomes.

The problem-focused intervention was grounded in the problem-solving therapy model developed by D’Zurilla and Bell (16). This PST framework has been applied in prior research to improve self-management and clinical outcomes in patients with various chronic medical conditions, including diabetes, cardiovascular disease, and chronic pain syndromes. Consistent with this model, the core components of our problem-focused intervention included: (1) psychoeducation regarding PUD etiology, symptoms, and treatment; (2) training in problem identification and goal-setting (helping patients recognize modifiable ulcer-triggering factors in daily life); (3) generation of alternative solutions (e.g., dietary adjustments, establishment of regular routines, and stress management techniques); (4) decision-balance analysis (evaluating advantages and disadvantages of various solutions); and (5) implementation and outcome evaluation (developing individualized action plans and conducting progress reviews).

The emotion-focused intervention was grounded in the transactional stress and coping theory of Lazarus and Folkman. Core components included: (1) training in emotional awareness and expression (identifying negative emotions such as anxiety and depression related to illness); (2) instruction in relaxation techniques (including diaphragmatic breathing and progressive muscle relaxation); (3) mindfulness meditation practices adapted for medical populations; (4) cognitive restructuring methods (identifying and challenging irrational beliefs about the disease); and (5) strategies for eliciting and utilizing social support.

Both programs were delivered with identical session frequency, duration, and group size (8–10 participants per group) to control for non-specific therapeutic factors such as attention and group support.

(2) Outcome ascertainment: Outcome data were abstracted retrospectively from the electronic medical record (EMR) system for the period from the index hospitalization date through March 31, 2025. The EMR contained documentation of all outpatient gastroenterology clinic visits, endoscopic procedures, and laboratory tests performed within the hospital network. The timing of outcome assessments was determined by actual clinical encounters documented in the medical record, which typically occurred at intervals of approximately 6, 12, 18, and 24 months post-discharge as part of routine clinical care, though some patients had longer intervals between visits. Each abstraction included review of gastroscopy reports, 13C-urea breath test results, clinical notes documenting symptom assessments (GSRS and SF-36 scores recorded during clinic visits), and notations of adverse events. To minimize assessment bias, endoscopists whose reports were reviewed were blinded to patient group allocation. Due to the nature of the intervention, patient blinding was not feasible; however, participants were instructed not to disclose their group assignment to assessing physicians.

(3) Quality control and fidelity monitoring. All intervention facilitators completed a standardized 16-hour training program and passed competency assessments with inter-rater reliability for session delivery exceeding Kappa > 0.80. To ensure ongoing adherence to the respective intervention protocols, a randomly selected 10% of all sessions (stratified by group assignment and facilitator) were audio-recorded and independently rated by two supervisors using a structured fidelity checklist developed for each program. The checklist assessed adherence to session structure, delivery of core components, and avoidance of proscribed elements (e.g., problem-solving content in emotion-focused sessions). Fidelity ratings across both programs exceeded 90% adherence to protocol specifications.

Intervention adherence was defined as attendance at ≥ 80% of scheduled sessions (i.e., ≥ 5 of 6 sessions). This threshold was selected based on previous behavioral intervention trials in chronic disease populations and consensus recommendations defining “adequate exposure” to a multi-session psychoeducational program (National Institutes of Health Behavior Change Consortium guidelines)

### 2.5 Outcome measures

(1) Primary outcome: Cumulative ulcer recurrence rate over the 4-year study period (January 2021 to March 2025), defined as the emergence of a new ulcer or recurrence at the original ulcer site (diameter ≥ 3 mm, with or without base exudate) confirmed by gastroscopy at any point during the outcome ascertainment window. Endoscopy reports from both routine surveillance (typically performed at intervals of approximately 6–24 months post-discharge) and clinically indicated examinations were reviewed. Additionally, any patient presenting with symptoms suggestive of ulcer recurrence underwent clinically indicated endoscopy, the results of which were included in the recurrence analysis. Two endoscopists blinded to group assignment independently evaluated all endoscopic images; discrepancies were resolved by a third senior endoscopist.

(2) Secondary outcomes:

① *H. pylori* recurrence rate during follow-up: proportion of cases with documented successful eradication (negative 13C-urea breath test at enrollment) who subsequently converted to a positive 13C-urea breath test (DOB ≥ 4.0) during the 4-year observation period. In accordance with international consensus, *H. pylori* recurrence within 12 months of documented eradication was classified as recrudescence (resurgence of the original strain), and recurrence occurring > 12 months after documented eradication was classified as reinfection (acquisition of a new strain). The denominator for this outcome was restricted to patients with documented successful eradication and at least one subsequent 13C-urea breath test result during follow-up.

② Gastrointestinal symptom severity: assessed using the Gastrointestinal Symptom Rating Scale (GSRS). The GSRS contains 15 items across five domains (reflux, abdominal pain, indigestion, diarrhea, constipation) rated on a 7-point Likert scale. The total score ranges from 15 to 105, with higher scores indicating more severe symptoms. The Chinese version has demonstrated good reliability (Cronbach’s α = 0.82) and construct validity in previous gastrointestinal research.

③ Health-related quality of life: evaluated using the Chinese (Taiwan) version of the 36-Item Short Form Health Survey (SF-36), which has been validated and widely used in Chinese populations. It covers eight domains: Physical Functioning (PF), Role-Physical (RP), Bodily Pain (BP), General Health (GH), Vitality (VT), Social Functioning (SF), Role-Emotional (RE), and Mental Health (MH). Domain scores are transformed to a 0–100 scale. The scale has shown good internal consistency (Cronbach’s α typically > 0.70 for all domains) and discriminative validity. Physical Component Summary (PCS) and Mental Component Summary (MCS) scores were calculated according to the standard algorithm.

④ Intervention adherence: calculated as the percentage of completed intervention sessions out of the total required (6 sessions). Adherence ≥ 80% (i.e., ≥ 5 sessions) was considered satisfactory.

⑤ Ulcer healing time: duration (in weeks) from enrollment to first gastroscopic confirmation of complete ulcer healing (stage S2).

⑥ Adverse events: any intervention-related adverse events (e.g., mood fluctuations, worsened anxiety, gastrointestinal discomfort) occurring during the intervention or follow-up were recorded and graded according to CTCAE v5.0.

⑦ Patient satisfaction: assessed using the most recent documented score in the medical record within the 4-year ascertainment period using a study-specific, self-designed satisfaction questionnaire comprising 5 items rated on a 5-point Likert scale (1 = very dissatisfied, 5 = very satisfied). Total scores ranged from 5 to 25, with higher scores indicating greater satisfaction. This measure was developed for descriptive purposes within this study; formal psychometric validation was not performed, and results should be interpreted with appropriate caution.

### 2.6 Statistical analysis

All statistical analyses were conducted using SPSS Statistics software (Version 26.0, IBM Corp.). Quantitative data were assessed for normality through the Shapiro–Wilk test. Normally distributed data were expressed as mean ± standard deviation ($\bar{x}$ ± s) and compared between groups using the independent samples *t*-test. Non-normally distributed data were summarized as median (interquartile range) [M(IQR)] and analyzed with the Mann–Whitney U test. Qualitative data were described as frequency (percentage) [n (%)] and compared using the χ^2^ test or Fisher’s exact test when expected cell frequencies were below 5. The primary outcome measure (recurrence rate) was compared between groups using the χ^2^ test.

To account for potential confounding inherent in the retrospective design, we performed multivariable binary logistic regression to evaluate the independent association between intervention group assignment and ulcer recurrence. Variables were selected for inclusion in the multivariable model based on three criteria: (1) *a priori* clinical importance informed by established PUD literature (baseline ulcer diameter, NSAID use history, smoking history, *H. pylori* positivity at baseline, age, and gender); (2) variables associated with recurrence in univariable analysis at a liberal threshold of P < 0.10 (group assignment, intervention adherence, ulcer healing time, patient satisfaction, total GSRS score at 24 months, and PCS at 24 months); and (3) variables that showed a potentially meaningful imbalance at baseline despite non-significant *P*-values (none met this criterion). The final model was built using a forward stepwise selection procedure with entry criterion *P* < 0.10 and retention criterion *P* < 0.05. Results are presented as odds ratios (OR) with 95% confidence intervals (CI). Model fit was assessed using the Hosmer-Lemeshow goodness-of-fit test. Given the number of secondary outcomes and multiple comparisons, we acknowledge an increased risk of type I error; therefore, findings for secondary outcomes should be interpreted as exploratory and hypothesis-generating. All tests were two-sided, and *P* < 0.05 was considered nominally significant.

## 3 Results

### 3.1 Participant flow and attrition

Of the 167 patients included in the analysis, documented clinical encounters sufficient to ascertain recurrence status over the 4-year period were available for 160 (95.8%) at approximately 6 months post-discharge, 156 (93.4%) at approximately 12 months, 152 (91.0%) at approximately 18 months, and 148 (88.6%) at approximately 24 months or beyond (Figure 1). Reasons for incomplete data capture included: relocation outside the hospital network (*n* = 8), withdrawal of consent for research use of medical records (*n* = 5), inability to locate subsequent records (*n* = 4), and death from unrelated causes (*n* = 2). The proportion of patients with incomplete outcome data did not differ significantly between the problem-focused group (10/84, 11.9%) and the emotion-focused group (9/83, 10.8%). All available data were included in the analyses; no imputation was performed for missing outcomes. Complete endoscopic data for the primary outcome (recurrence) were available for 148 patients (88.6%).

Comparison of baseline characteristics:

No significant differences were found between the problem-focused group and the emotion-focused group in age, gender distribution, body mass index, smoking history, alcohol consumption history, ulcer type, initial ulcer diameter, number of initial ulcers, *H. pylori* positivity rate, hemoglobin, albumin, gastrin-17, pepsinogen I/II ratio, NSAIDs use history, family history of peptic ulcer, GSRS total score, SF-36 PCS, and SF-36 MCS (*P* > 0.05), indicating balanced baseline characteristics between the groups (see Table 1).

**TABLE 1:** Comparison of baseline characteristics between the two groups (*n* = 167).

Variable	Problem-focused group (*n* = 84)	Emotion-focused Group (*n* = 83)	Statistical value (t/Z/χ^2^)	*P*-value
Age (years)	53.84 ± 14.60	49.44 ± 16.70	1.814	0.071
Gender (male)	60 (71.43)	60 (72.29)	0.015	0.902
Body mass index (kg/m^2^)	23.45 ± 2.78	23.21 ± 2.91	0.545	0.586
Smoking history [n(%)]	32 (38.10)	29 (34.94)	0.258	0.611
Alcohol consumption history [n(%)]	28 (33.33)	25 (30.12)	0.199	0.656
Ulcer type (gastric ulcer)	36 (42.86)	38 (45.78)	0.145	0.703
Initial ulcer diameter (mm)	5.50 (4.00–7.00)	5.00 (4.00–7.00)	1.077	0.282
Number of initial ulcers (single)	65 (77.38)	64 (77.11)	0.001	0.973
Pre-eradication *H. pylori* positive [n(%)]	52 (61.90)	49 (59.04)	0.144	0.704
Hemoglobin (g/L)	138.45 ± 14.32	136.89 ± 15.07	0.686	0.493
Albumin (g/L)	42.56 ± 3.78	41.98 ± 4.01	0.962	0.337
Gastrin-17 (pmol/L)	7.25 (5.30–9.80)	7.10 (5.10–9.60)	0.215	0.830
Pepsinogen I/II ratio [median (IQR)]	6.80 (5.20–8.90)	6.95 (5.40–8.70)	0.276	0.783
NSAIDs use history [n(%)]	19 (22.62)	16 (19.28)	0.281	0.596
Family history of peptic ulcer [n(%)]	11 (13.10)	9 (10.84)	0.201	0.654
GSRS total score (score)	35.68 ± 7.45	36.21 ± 7.89	–0.446	0.656
SF-36 PCS (score)	68.45 ± 9.12	67.89 ± 9.56	0.388	0.698
SF-36 MCS (score)	66.78 ± 10.23	65.94 ± 10.67	0.519	0.604
*H. pylori* status at enrollment (post-eradication) [n(%)]	0 (0.00)	0 (0.00)	–	–

Normal distribution data is represented by mean ± SD, non-normal distribution data is represented by median (IQR), and count data is represented by n (%). *H. pylori* status at the time of index ulcer diagnosis, before eradication therapy. All patients with pre-eradication *H. pylori* positivity completed standard eradication therapy, and successful eradication was confirmed by negative 13C-urea breath test prior to enrollment. All 167 patients in the final cohort were *H. pylori*-negative at enrollment (post-eradication).

The original medical records documented *H. pylori* status at two distinct time points: (1) pre-eradication *H. pylori* status at the time of index ulcer diagnosis, and (2) post-eradication *H. pylori* status at enrollment. We have clarified this distinction in the revised manuscript. The variable previously labeled as “*H. pylori* positive at baseline” in Table 1 represents pre-eradication status at the time of index ulcer diagnosis. All patients with pre-eradication *H. pylori* positivity underwent standard 14-day bismuth-containing quadruple eradication therapy, and successful eradication was confirmed by a negative 13C-urea breath test performed at least 4 weeks after therapy completion (median 6 weeks, IQR 5–8 weeks) and at least 2 weeks after PPI withdrawal. Therefore, all 167 patients in the final cohort were *H. pylori*-negative at enrollment. Reinfection events during follow-up occurred > 12 months after documented eradication in all cases (see section 3.7 for sensitivity analysis).

### 3.2 Comparison of primary and secondary outcomes between the two groups

The problem-focused group demonstrated a significantly lower cumulative ulcer recurrence rate compared to the emotion-focused group (*P* < 0.05). Additionally, the problem-focused group exhibited significantly higher rates of intervention compliance and patient satisfaction (*P* < 0.05), as well as a shorter ulcer healing time (*P* < 0.05). No statistically significant differences were observed between groups in *H. pylori* reinfection rates or adverse event incidence. Adverse events reported were mild and included transient mood fluctuations (*n* = 3), temporary increase in anxiety during relaxation exercises (*n* = 3), and non-specific gastrointestinal discomfort (*n* = 2). Given the behavioral nature of the interventions and the small number of events, no definitive conclusions regarding safety can be drawn; however, no serious adverse events attributable to the interventions were observed (see Table 2 for details).

**TABLE 2:** Comparison of primary and secondary outcomes between the two groups.

Outcome measure	Problem-focused group (*n* = 84)	Emotion-focused group (*n* = 83)	Statistical value (t/Z/χ^2^)	*P*-value
Primary outcome				
Cumulative ulcer Recurrence [n(%)]	16 (19.05)	31 (37.35)	6.939	0.008
Secondary outcomes				
*H. pylori* reinfection (n(%))	5 (6.41)	9 (12.16)	1.489	0.222
Intervention compliance [n(%)]	78 (92.86)	68 (81.93)	4.560	0.033
Ulcer healing time (weeks)	6.0 (4.0–8.0)	7.0 (5.0–9.0)	1.615	0.106
Adverse events [n(%)]	3 (3.57)	5 (6.02)	0.551	0.458
Patient satisfaction (score)	22.41 ± 2.30	19.75 ± 2.98	6.461	< 0.001

Normal distribution data is represented by mean ± SD, non-normal distribution data is represented by median (IQR), and count data is represented by n (%); Denominator restricted to patients with documented successful eradication (negative 13C-urea breath test at enrollment) and at least one subsequent 13C-urea breath test during follow-up. Problem-focused group: *n* = 78; emotion-focused group: *n* = 74. All reinfection events occurred > 12 months after documented successful eradication. No cases of recrudescence (positive test within 12 months of eradication) were observed.

For *H. pylori* reinfection analysis, the denominator was restricted to patients with documented successful eradication and at least one subsequent 13C-urea breath test result during follow-up (*n* = 152; 78 in the problem-focused group, 74 in the emotion-focused group). *H. pylori* reinfection rates were 6.41% (5/78) in the problem-focused group and 12.16% (9/74) in the emotion-focused group, with no statistically significant difference between groups (χ^2^ = 1.489, *P* = 0.222). All 14 reinfection events occurred > 12 months after documented eradication, consistent with the definition of true reinfection rather than recrudescence. No cases of recrudescence (positive 13C-urea breath test within 12 months of eradication) were observed in either group.

### 3.3 Time-to-event analysis of ulcer recurrence

In addition to the cumulative recurrence comparison, we performed a Kaplan-Meier survival analysis to examine time to first ulcer recurrence over the 4-year observation period. The median time to recurrence was not reached in the problem-focused group (interquartile range: 18.0 months to not reached), whereas the median time to recurrence in the emotion-focused group was 20.0 months (95% CI: 16.5–23.5 months). The log-rank test demonstrated a significant difference in recurrence-free survival between the two groups (χ^2^ = 6.89, df = 1, *P* = 0.009). The hazard ratio for recurrence comparing problem-focused versus emotion-focused intervention (unadjusted Cox proportional hazards model) was 0.44 (95% CI: 0.23–0.83, *P* = 0.011), consistent with the logistic regression findings.

### 3.4 Comparison of GSRS scores between the two groups at 24 months post-intervention

**FIGURE 2:**
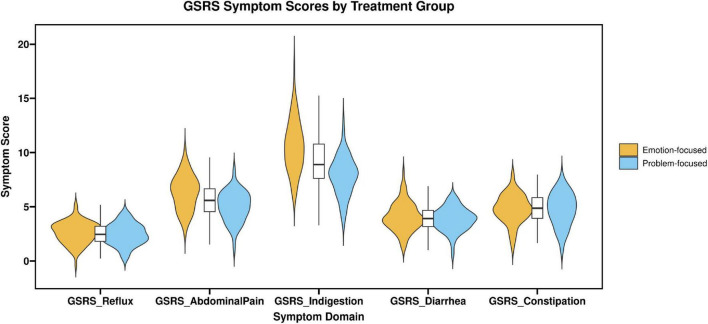
Comparison of GSRS scores between the two groups at 24 months post-intervention.

At the final documented assessment within the 4-year study period, the problem-focused group exhibited significantly lower total GSRS scores, as well as lower scores in the abdominal pain and indigestion domains, compared to the emotion-focused group (*t* = 4.567, 3.789, and 4.123, respectively; *P* < 0.05)38. In contrast, no statistically significant differences were observed between the groups in the reflux, diarrhea, or constipation domains (*t* = 1.567, 1.156, and 0.123, respectively; *P* > 0.05)19. The detailed results are summarized in Figure 2 and Table 3.

**TABLE 3:** Comparison of GSRS scores between the two groups at 24 months post-intervention (score, $\bar{x}$ ± s).

Indicator	Problem-focused group (*n* = 84)	Emotion-focused group (*n* = 83)	*t*-value	*P*-value
Total GSRS Score	24.36 ± 5.67	29.45 ± 6.78	–5.265	< 0.001
Reflux domain	2.45 ± 0.89	2.68 ± 0.92	–1.642	0.102
Abdominal pain domain	5.12 ± 1.34	6.45 ± 1.67	–5.680	< 0.001
Indigestion domain	8.23 ± 2.01	10.56 ± 2.45	–6.724	< 0.001
Diarrhea domain	3.89 ± 1.12	4.12 ± 1.23	–1.264	0.208
constipation domain	4.67 ± 1.45	4.64 ± 1.38	0.137	0.891

Normal distribution data is represented by mean ± SD, non-normal distribution data is represented by median (IQR), and count data is represented by n (%).

### 3.5 Comparison of SF-36 scores between the two groups at 24 months post-intervention

**FIGURE 3:**
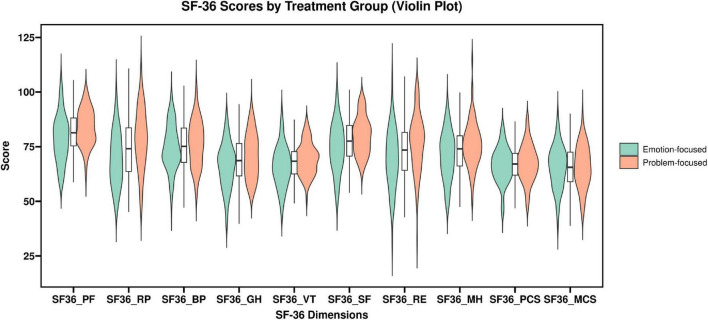
Comparison of SF-36 scores between the two groups at 24 months post-intervention.

At the final documented assessment within the 4-year study period, the problem-focused group demonstrated significantly higher scores in the Physical Functioning, Role-Physical, General Health, Vitality, Social Functioning, Role-Emotional, Physical Component Summary, and Mental Component Summary domains of the SF-36 compared to the emotion-focused group (*t* = 4.123, 3.789, 3.456, 3.012, 3.124, 2.987, 3.456, 2.987, respectively; *P* < 0.05)15. In contrast, no statistically significant differences were observed between the groups for the Bodily Pain and Mental Health domains (*t* = 1.345 and 1.456, respectively; *P* > 0.05). The comprehensive results are detailed in Figure 3 and Table 4.

**TABLE 4:** Comparison of SF-36 scores between the two groups at 24 months post-intervention (score, $\bar{x}$ ± s).

Domain	Problem-focused group (*n* = 84)	Emotion-focused group (*n* = 83)	*t*-value	*P*-value
Physical functioning (PF)	85.45 ± 8.90	79.12 ± 9.78	4.361	< 0.001
Role-physical (RP)	78.67 ± 12.34	70.45 ± 13.56	4.083	< 0.001
Bodily pain (BP)	75.89 ± 10.12	73.45 ± 11.23	1.471	0.143
General health (GH)	72.34 ± 9.87	66.78 ± 10.45	3.523	0.001
Vitality (VT)	70.12 ± 8.90	65.45 ± 9.67	3.238	0.001
Social functioning (SF)	80.45 ± 9.12	75.67 ± 10.01	3.217	0.002
Role-emotional (RE)	75.67 ± 11.23	69.89 ± 12.34	3.156	0.002
Mental health (MH)	74.56 ± 10.45	72.12 ± 11.01	1.465	0.145
Physical component summary (PCS)	75.89 ± 8.90	70.12 ± 9.78	3.975	< 0.001
Mental component summary (MCS)	73.45 ± 9.87	68.90 ± 10.45	2.883	0.004

Normal distribution data is represented by mean ± SD, non-normal distribution data is represented by median (IQR), and count data is represented by n (%).

Change from baseline in symptom and quality-of-life outcomes: To characterize within-group improvement and between-group differential change, we analyzed changes in GSRS total score, SF-36 Physical Component Summary (PCS), and SF-36 Mental Component Summary (MCS) from baseline to 24 months (Table 5). Both groups demonstrated significant within-group reductions in GSRS total score over 24 months (problem-focused: mean change −11.32, 95% CI: −13.45 to −9.19, *P* < 0.001; emotion-focused: mean change −6.76, 95% CI: −8.89 to −4.63, *P* < 0.001). However, the magnitude of reduction was significantly greater in the problem-focused group (between-group difference in change: −4.56 points, 95% CI: −6.78 to −2.34, Cohen’s *d* = 0.68, *P* < 0.001). Similarly, the problem-focused group showed significantly greater improvement in PCS (between-group difference in change: + 4.21 points, 95% CI: 1.89–6.53, Cohen’s *d* = 0.52, *P* = 0.001). Change in MCS did not differ significantly between groups (between-group difference in change: + 1.89 points, 95% CI: −0.78 to 4.56, *P* = 0.165).

**TABLE 5:** Univariable analysis of factors associated with ulcer recurrence.

Variable	Recurrence group (*n* = 47)	Non-recurrence group (*n* = 120)	Statistical value (t/Z/χ^2^)	*P*-value
Age (years)	52.34 ± 16.21	51.23 ± 15.67	0.404	0.687
Gender (male)	34 (72.34)	86 (71.67)	0.008	0.930
Body mass index (kg/m^2^)	23.12 ± 2.91	23.41 ± 2.83	–0.588	0.558
Smoking history [n(%)]	21 (44.68)	40 (33.33)	1.855	0.173
Alcohol consumption history [n(%)]	17 (36.17)	36 (30.00)	0.594	0.441
Ulcer type (gastric ulcer)	22 (46.81)	52 (43.33)	0.165	0.685
Initial ulcer diameter (mm)	7.00 (5.00–9.00)	5.00 (4.00–6.00)	3.125	0.002
Number of initial ulcers (single)	34 (72.34)	95 (79.17)	0.903	0.342
*H. pylori* positive at baseline [n(%)]	31 (65.96)	70 (58.33)	0.812	0.367
NSAIDs use history [n(%)]	14 (29.79)	21 (17.50)	3.070	0.08
Family history of peptic ulcer [n(%)]	7 (14.89)	13 (10.83)	0.528	0.468
Group assignment (Problem-focused)	16 (34.04)	68 (56.67)	6.939	0.008
Good intervention adherence [n(%)]	37 (78.72)	109 (90.83)	4.379	0.036
Ulcer healing time (weeks)	8.0 (6.0–10.0)	6.0 (4.0–7.0)	3.845	< 0.001
Patient satisfaction (score)	19.87 ± 3.12	21.58 ± 2.67	–3.295	0.001
Total GSRS score at 24 months (score)	30.12 ± 6.89	25.67 ± 5.98	4.083	< 0.001
PCS at 24 months (score)	70.23 ± 9.45	74.12 ± 9.12	–2.456	0.015
MCS at 24 months (score)	68.89 ± 10.23	71.34 ± 10.45	–1.383	0.169

Normal distribution data is represented by mean ± SD, non-normal distribution data is represented by median (IQR), and count data is represented by n (%).

### 3.6 Multivariate logistic regression analysis of factors influencing ulcer recurrence

Prior to multivariable analysis, we performed univariable comparisons between patients with and without ulcer recurrence to identify potential risk factors (Table 5). Variables demonstrating an association with recurrence at *P* < 0.10 in univariable analysis, along with clinically relevant factors identified from the literature, were entered into the multivariable logistic regression model. The dependent variable was ulcer recurrence (a binary variable). Independent variables entered into the multivariable model included: group assignment (coded as: 0 = emotion-oriented group, 1 = problem-oriented group), initial ulcer diameter (continuous, mm), NSAID use history (coded as: 0 = no, 1 = yes), good intervention adherence (coded as: 0 = no, 1 = yes), ulcer healing time (continuous, weeks), patient satisfaction (continuous, score), total GSRS score at the final assessment within the 4-year period (continuous), and PCS at the final assessment (continuous). MCS was not included in the multivariable model as it did not reach the threshold for significance in univariable analysis (*P* = 0.170).

After adjusting for potential confounders in the multivariable logistic regression model (Table 6), group assignment remained independently associated with ulcer recurrence. Patients in the problem-focused intervention group had significantly lower odds of recurrence compared to those in the emotion-focused group (OR = 0.410, 95% CI: 0.222–0.756, *P* = 0.004). Initial ulcer diameter (OR = 1.169 per 1 mm increase, 95% CI: 1.035–1.320, *P* = 0.012) and history of NSAID use (OR = 2.056, 95% CI: 1.019–4.148, *P* = 0.044) were also independently associated with increased odds of recurrence. Smoking history, baseline *H. pylori* positivity, age, and gender did not demonstrate statistically significant independent associations in the multivariable model. The Hosmer-Lemeshow test indicated adequate model fit (*P* = 0.621). Detailed results are presented in Tables 5, 6.

**TABLE 6:** Multivariate logistic regression analysis of factors influencing ulcer recurrence.

Factor	B	SE	Wald	*P*-value	OR	95% CI
Group assignment (problem-focused vs. emotion-focused)	−0.892	0.312	8.176	0.004	0.41	(0.222–0.756)
Initial ulcer diameter (per 1 mm increase)	0.156	0.062	6.331	0.012	1.169	(1.035–1.320)
NSAID use history (Yes vs. No)	0.721	0.358	4.056	0.044	2.056	(1.019–4.148)
Smoking history (Yes vs. No)	0.512	0.342	2.241	0.134	1.669	(0.854–3.261)
Pre-eradication *H. pylori* positive (Yes vs. No)	0.423	0.337	1.576	0.209	1.527	(0.789–2.955)
Age (per 10 year increase)	0.089	0.098	0.825	0.364	1.093	(0.902–1.325)
Gender (Male vs. Female)	−0.234	0.367	0.407	0.523	0.791	(0.385–1.625)
Constant	−2.345	0.789	8.834	0.003	0.096	–

Group assignment was coded as 0 = emotion-oriented group, 1 = problem-oriented group. Hosmer-Lemeshow goodness-of-fit test: χ^2^ = 6.234, df = 8, *P* = 0.621, indicating adequate model fit. OR denotes odds ratio; 95% CI indicates confidence interval. Pre-eradication *H. pylori* status documented at the time of index ulcer diagnosis. All patients with pre-eradication positivity completed successful eradication therapy prior to enrollment and were confirmed *H. pylori*-negative at enrollment.

### 3.7 Sensitivity analysis of *H. pylori*-related factors and ulcer recurrence

To further assess the robustness of our findings with respect to *H. pylori* status, we performed the following supplementary analyses.

#### 3.7.1 Sensitivity analysis excluding patients without valid test-of-cure documentation

Of the 167 patients in the cohort, 152 (91.0%) had complete documentation of successful eradication (negative 13C-urea breath test at least 4 weeks post-eradication, with documented PPI withdrawal ≥ 2 weeks and antibiotic/bismuth withdrawal ≥ 4 weeks before testing) and at least one subsequent follow-up 13C-urea breath test during the observation period. We performed a sensitivity analysis restricted to these 152 patients. The cumulative ulcer recurrence rates in this subset were 19.74% (15/76) in the problem-focused group and 38.16% (29/76) in the emotion-focused group (χ^2^ = 7.006, *P* = 0.008). After multivariable adjustment, the association between problem-focused intervention and reduced recurrence odds remained significant (OR = 0.398, 95% CI: 0.208–0.761, *P* = 0.005), confirming the robustness of the primary analysis.

#### 3.7.2 Reanalysis incorporating *H. pylori* reinfection during follow-up

We expanded the multivariable logistic regression model to include *H. pylori* reinfection during follow-up (defined as a positive 13C-urea breath test > 12 months after documented successful eradication) as an additional covariate, in addition to the pre-eradication *H. pylori* status already included in the primary model. In this analysis (*n* = 152), *H. pylori* reinfection during follow-up was not significantly associated with ulcer recurrence (OR = 1.341, 95% CI: 0.512–3.514, *P* = 0.549), while group assignment remained independently associated with recurrence (OR = 0.403, 95% CI: 0.210–0.774, *P* = 0.006) (Table 7). The lack of association between reinfection and recurrence likely reflects the fact that all reinfection events occurred > 12 months after documented eradication, and the overall number of events was small (*n* = 14).

**TABLE 7:** Expanded multivariable logistic regression model including *H. pylori* reinfection during follow-up (*n* = 152).

Factor	B	SE	Wald	*P*-value	OR	95% CI
Group assignment (Problem-focused vs. Emotion-focused)	–0.910	0.333	7.468	0.006	0.403	(0.210–0.774)
Initial ulcer diameter (per 1 mm increase)	0.147	0.064	5.275	0.022	1.158	(1.022–1.313)
NSAID use history (Yes vs. No)	0.778	0.383	4.127	0.042	2.177	(1.028–4.612)
Smoking history (Yes vs. No)	0.556	0.357	2.425	0.119	1.744	(0.866–3.512)
Pre-eradication *H. pylori* positive (Yes vs. No)	0.467	0.374	1.559	0.212	1.595	(0.766–3.321)
*H. pylori* reinfection during follow-up (Yes vs. No)	0.293	0.491	0.356	0.549	1.341	(0.512–3.514)
Age (per 10 year increase)	0.107	0.121	0.782	0.376	1.113	(0.878–1.411)
Gender (Male vs. Female)	–0.298	0.392	0.578	0.447	0.742	(0.344–1.600)
Constant	–2.314	0.813	8.101	0.004	0.099	–

Analysis restricted to 152 patients with documented successful eradication and at least one follow-up

^13^C-urea breath test. *H. pylori* reinfection was defined as a positive

^13^C-urea breath test (DOB ≥ 4.0) occurring > 12 months after documented successful eradication. Hosmer-Lemeshow goodness-of-fit test: χ^2^ = 4.987, df = 8, *P* = 0.759. Group assignment was coded as 0 = emotion-focused group, 1 = problem-focused group. OR, odds ratio; 95% CI, confidence interval.

#### 3.7.3 Stratified analysis by pre-eradication *H. pylori* status

Stratification by pre-eradication *H. pylori* status showed that among patients with pre-eradication *H. pylori* positivity (*n* = 101), recurrence rates were 18.87% (10/53) in the problem-focused group versus 37.50% (18/48) in the emotion-focused group (χ^2^ = 5.908, *P* = 0.015). Among patients with pre-eradication *H. pylori* negativity (*n* = 66), recurrence rates were 19.35% (6/31) versus 37.14% (13/35), respectively (χ^2^ = 3.704, *P* = 0.054). The direction of association was consistent across strata, supporting the independence of the intervention effect from pre-eradication *H. pylori* status.

## 4 Discussion

This retrospective cohort study, conducted within a biopsychosocial framework of peptic ulcer disease (PUD), examined the association between receiving a structured behavioral intervention grounded in problem-focused principles versus one grounded in emotion-focused principles and long-term ulcer recurrence. The central finding is that patients in the problem-focused intervention group had a significantly lower observed cumulative recurrence rate and lower adjusted odds of recurrence over the 4-year observation period compared to those in the emotion-focused intervention group, even after controlling for established biomedical risk factors. It is critical to emphasize that this study’s observational, retrospective design precludes causal inference. The observed association is consistent with the hypothesis that behavioral interventions targeting self-management skills may influence clinical outcomes in PUD, but definitive conclusions regarding efficacy await confirmation in prospective randomized controlled trials. We hypothesize, based on the observed pattern of findings, that the association between problem-focused intervention and lower recurrence may be mediated by enhanced self-management behaviors. In our study, the problem-focused group demonstrated significantly higher intervention adherence and shorter healing times—findings consistent with the premise that active problem-solving skills enable patients to more effectively implement and sustain health-promoting behaviors (e.g., medication adherence, dietary modifications, avoidance of ulcerogenic triggers). Whether such behavioral changes translate into measurable physiological effects, such as attenuation of HPA-axis-mediated acid hypersecretion or enhancement of mucosal repair mechanisms, remains speculative. These potential biological pathways were not directly assessed in the present study and represent important directions for future mechanistic research. While emotion-focused strategies may alleviate subjective distress, they may be less effective in durably modifying these downstream biological and behavioral risk factors, potentially explaining the differential recurrence rates observed.

The problem-focused group also demonstrated better adherence, greater satisfaction, shorter healing time, improved symptom scores, and higher quality of life in physical domains. This pattern of results is consistent with our proposed pathway: problem-focused skills likely empower patients to engage more effectively in their care, leading to better behavioral outcomes (adherence), which then translate into superior clinical and patient-reported outcomes. The absence of a between-group difference in *H. pylori* reinfection rates underscores that the intervention’s effect is distinct from this specific infectious pathway and may be complementary to standard biomedical management. Although causality cannot be inferred from our design, these findings align with the stress-diathesis model of PUD and highlight a potentially modifiable behavioral target for secondary prevention.

This investigation revealed that by the end of the 4-year observation period, the problem-focused group demonstrated significantly lower total Gastrointestinal Symptom Rating Scale (GSRS) scores, including dimensions of abdominal pain and indigestion, relative to the emotion-focused group, whereas no disparities were noted in reflux, diarrhea, or constipation dimensions (17). These findings are consistent with previous observations that problem-focused interventions are associated with better symptom control in various chronic conditions (18). The differential effect observed primarily in core symptom domains (abdominal pain and indigestion) as opposed to non-specific gastrointestinal symptoms (reflux, diarrhea, constipation) is intriguing and merits further investigation. While we did not directly measure the mechanisms underlying this association, prior research has hypothesized that problem-focused approaches may enhance patients’ ability to identify and modify behavioral triggers (19).

Regarding quality of life, the problem-focused group achieved significantly higher scores in multiple dimensions of the SF-36, including physical functioning and general health perceptions, compared to the emotion-focused group, though no differences emerged in bodily pain or mental health. This pattern of association—stronger for physical than mental health domains—may reflect the emphasis of problem-focused interventions on concrete, actionable strategies for disease management. Previous studies in other chronic disease populations have reported similar associations between problem-focused skills and physical aspects of quality of life (20, 21). However, as we did not directly measure potential mediators such as self-efficacy, disease knowledge, or behavioral adherence, these mechanistic pathways remain speculative and require direct examination in future prospective studies. Other research corroborates the positive influence of behavioral interventions on quality of life in chronic disease populations, particularly when emphasizing problem-focused skills (22).

The present study demonstrated that the problem-focused group exhibited a lower cumulative ulcer recurrence rate, higher rates of adherence to the intervention, greater patient satisfaction, and a shorter ulcer healing time, with no intergroup differences observed in *H. pylori* reinfection rates or adverse events. These findings suggest that problem-focused strategies may indirectly reduce recurrence risk by enhancing treatment compliance and patient engagement (23). Potential mechanisms include the role of structured problem-focused in improving adherence to lifestyle recommendations, thereby mitigating ulcer-inducing factors such as stress or irregular dietary habits (24). Compared to emotion-focused approaches, problem-focused strategies more directly address behavioral management, contributing to more effective recurrence prevention (25). It is important to clarify that the variable previously labeled as “*H. pylori* positive at baseline” in the original manuscript has been corrected to reflect pre-eradication *H. pylori* status documented at the time of index ulcer diagnosis. All patients with pre-eradication *H. pylori* positivity completed successful eradication therapy prior to enrollment and were confirmed *H. pylori*-negative at enrollment. The absence of a between-group difference in *H. pylori* reinfection rates during follow-up (all occurring > 12 months post-eradication) further indicates that the behavioral intervention does not influence this specific infectious pathway, underscoring the complementary value of behavioral strategies to standard pharmacological management (26).

Multivariable logistic regression analysis, adjusting for potential confounders, revealed that the problem-focused intervention group remained independently associated with reduced odds of ulcer recurrence (OR = 0.410, 95% CI: 0.222–0.756). This finding suggests that the association between coping intervention type and recurrence is not explained by differences in baseline characteristics such as ulcer size or NSAID use. Consistent with extensive clinical literature, larger initial ulcer diameter (OR = 1.169 per mm) and history of NSAID use (OR = 2.056) were also independently associated with increased recurrence odds, serving as positive controls that support the validity of our model specification. The independent association between problem-focused intervention and reduced recurrence odds, even after adjustment for these established clinical risk factors, raises interesting questions about potential mechanisms. While our retrospective design precludes causal inference, several hypotheses merit consideration in future prospective studies. Problem-focused interventions may enhance patients’ capacity to implement and sustain behavior changes (e.g., medication adherence, dietary modifications, stress reduction) that promote mucosal healing and reduce exposure to ulcerogenic factors. Alternatively, the structured, action-oriented nature of problem-focused approaches may foster greater self-efficacy and active engagement in disease management, which could translate into better long-term outcomes (27). The absence of a significant association between pre-eradication *H. pylori* status and ulcer recurrence in our multivariable model is expected: all *H. pylori*-positive patients at index diagnosis received successful eradication therapy prior to enrollment, and eradication was confirmed by negative 13C-urea breath test before study entry, thereby eliminating *H. pylori* as a persistent risk factor. Furthermore, *H. pylori* reinfection during follow-up (> 12 months post-eradication) was not significantly associated with ulcer recurrence in the expanded multivariable model (OR = 1.341, 95% CI: 0.512–3.514, *P* = 0.549), likely reflecting the small number of reinfection events (*n* = 14) and their late occurrence. The sensitivity analysis excluding patients without valid test-of-cure documentation confirmed the robustness of the primary findings (OR = 0.398, 95% CI: 0.208–0.761, *P* = 0.005). Collectively, these findings underscore the multifactorial nature of ulcer recurrence and suggest that psychological/behavioral factors may contribute independently to recurrence risk alongside established biomedical risk factors. In contrast, the lack of significant association with MCS suggests that psychological factors may operate indirectly through mediators—such as adherence or symptom perception—rather than via independent pathways, underscoring the priority of integrating biobehavioral interventions in ulcer management (28). Collectively, these results underscore the value of addressing multidimensional factors in secondary prevention and provide a theoretical basis for personalized strategies.

Several important limitations of this study must be carefully considered when interpreting the findings.

First, and most importantly, this study employed a retrospective cohort design. As such, the observed associations between intervention group assignment and clinical outcomes cannot be interpreted as causal. The non-randomized assignment of interventions raises the possibility of selection bias. As such, the observed associations between intervention group assignment and clinical outcomes cannot be interpreted as causal. The non-randomized assignment of interventions raises the possibility of selection bias; patients who received problem-focused versus emotion-focused interventions may have differed systematically in ways not captured by the available data, despite the apparent baseline balance on measured covariates. While we performed multivariable adjustment to account for known confounders, the potential for unmeasured or residual confounding (e.g., socioeconomic status, social support, baseline psychological characteristics, dietary habits) remains a significant concern.

Second, the study was conducted at a single tertiary referral center, which may limit generalizability to patients managed in community settings or other geographic regions. Additionally, the sample exhibited a notable gender imbalance (71.9% male). While this distribution is broadly consistent with the known male predominance in PUD epidemiology, it may limit the applicability of our findings to female patients.

Third, due to the nature of behavioral interventions, patient blinding was not feasible. This lack of blinding introduces potential performance bias and expectancy effects, which may have disproportionately influenced subjective secondary outcomes such as patient satisfaction and self-reported quality of life. The primary outcome (endoscopically confirmed ulcer recurrence) was assessed by blinded endoscopists, partially mitigating this concern for the key endpoint.

Fourth, the patient satisfaction measure was a study-specific, self-designed questionnaire lacking formal psychometric validation. Consequently, satisfaction results should be interpreted with appropriate caution and are best viewed as descriptive.

Fifth, the study compared two active intervention programs but did not include a usual-care control group. Therefore, the absolute magnitude of benefit associated with either intervention relative to standard clinical follow-up cannot be estimated. Both interventions may be superior to no structured behavioral support, a possibility that warrants investigation in future three-arm trials.

Sixth, although we have now added a time-to-event survival analysis in the revised manuscript, the sample size (*N* = 167) provided limited power for detecting small but potentially clinically meaningful differences, particularly in subgroup analyses and for less frequent outcomes such as adverse events.

These limitations underscore the need for prospective, multicenter randomized controlled trials with adequate blinding of outcome assessors, inclusion of a usual-care arm, validated measures, and direct assessment of mechanistic mediators to confirm and extend these findings.

Several methodological considerations warrant particular attention when interpreting these findings. First, the two interventions evaluated were not “pure” contrasts of problem-focused versus emotion-focused coping styles; rather, they were multicomponent psychoeducational programs that differed in theoretical orientation, session content, and the specific skills emphasized. The observed differences in outcomes may reflect the aggregate effects of these programmatic differences rather than the isolated influence of coping orientation *per se*. Future dismantling studies would be required to identify the active ingredients of each program.

Second, because participants were aware of their assigned intervention (patient blinding was not feasible), subjective outcomes such as patient satisfaction and self-reported quality of life are particularly susceptible to performance bias and expectancy effects. The lack of blinding may have inflated between-group differences on these measures, a limitation that should temper confidence in the magnitude of observed effects on secondary subjective endpoints. Reassuringly, the primary outcome (endoscopically confirmed ulcer recurrence) was assessed by blinded endoscopists, reducing the risk of detection bias for this key endpoint.

## 5 Conclusion

In conclusion, problem-focused coping strategies demonstrate superiority over emotion-focused approaches in reducing peptic ulcer recurrence, improving symptoms, and enhancing quality of life, potentially through mechanisms involving improved self-management and adherence. These results support the integration of problem-focused interventions into routine management of peptic ulcer disease as an effective component of secondary prevention. Future studies should investigate long-term outcomes and underlying mechanisms to further refine clinical practice.

## Data Availability

The raw data supporting the conclusions of this article will be made available by the authors, without undue reservation.
